# Investigating the Causes of an Extinction Catastrophe: Controlling Introduced Predators Remains Essential for Conserving Australia’s Mammals

**DOI:** 10.1093/biosci/biaf204

**Published:** 2026-01-21

**Authors:** John C Z Woinarski, Sarah M Legge, Katherine Moseby, Andrew A Burbidge, Alexandra J R Carthey, Chris R Dickman, Tim S Doherty, Jason Ferris, Diana O Fisher, Matthijs Hollanders, Bronwyn A Hradsky, Chris N Johnson, Chris J Jolly, John Kanowski, Mike Letnic, Rachel T Mason, Hugh McGregor, Brett P Murphy, Reece Pedler, John L Read, Anthony R Rendall, Alyson Stobo-Wilson, Jonathan Webb, Bruce L Webber, Rebecca West, Euan G Ritchie

**Affiliations:** Research Institute for the Environment and Livelihoods, Charles Darwin University, Casuarina, Northern Territory, 0909, Australia; Research Institute for the Environment and Livelihoods, Charles Darwin University, Casuarina, Northern Territory, 0909, Australia; Fenner School of Society and the Environment, The Australian National University, Acton, Canberra, Australian Capital Territory, 2601, Australia; Centre for Ecosystem Science, School of Biological, Earth and Environmental Sciences, University of New South Wales, Kensington, Sydney, 2033, Australia; Floreat, Western Australia 6104, Australia; School of Natural Sciences, Macquarie University, North Ryde, NSW 2109, Australia; School of Life and Environmental Sciences, The University of Sydney, Sydney, NSW 2006, Australia; Biodiversity and Conservation Science, Department of Biodiversity, Conservation and Attractions, Kings Park, Perth, WA 6005, Australia; Department of Climate Change, Energy, the Environment and Water, Canberra ACT, 2600, Australia; School of the Environment, University of Queensland, St Lucia, Queensland 4072, Australia; College of Engineering, Science and Environment, School of Environmental and Life Sciences, University of Newcastle, Newcastle, NSW, 2308, Australia; Quantitative and Applied Ecology, School of Agriculture, Food and Ecosystem Sciences, University of Melbourne, Parkville, VIC 3010, Australia; School of Natural Sciences, University of Tasmania, PO Box 805 Sandy Bay 7006, Hobart, Tasmania, Australia; School of Natural Sciences, Macquarie University, Sydney, NSW, Australia; Research Institute for the Environment and Livelihoods, Charles Darwin University, NT 0909, Australia; Australian Wildlife Conservancy, Subiaco, WA 6008, Australia; Evolution and Ecology Research Centre, University of New South Wales, Sydney 2052, Australia; School of Biological Sciences, Monash University, Melbourne, Victoria, Australia, 3800; Nature Foundation, Prospect, South Australia, 5082, Australia; Research Institute for the Environment and Livelihoods, Charles Darwin University, Casuarina, Northern Territory, 0909, Australia; Centre for Ecosystem Science, School of Biological, Earth and Environmental Sciences, University of New South Wales, Kensington, Sydney, 2033, Australia; University of Adelaide, Adelaide, 5005, SA, Australia; School of Life and Environmental Sciences, Deakin University, Burwood, Victoria 3125, Australia; Australian Wildlife Conservancy, Subiaco, WA 6008, Australia; Research Institute for the Environment and Livelihoods, Charles Darwin University, Casuarina, NT 0909, Australia; School of Life Sciences, The University of Technology Sydney, Broadway, NSW 2007, Australia; Bush Heritage Australia, Melbourne, Victoria 3008, Australia; and School of Biological Sciences, University of Western Australia, Crawley, Western Australia 6009, Australia; School of Biological, Earth and Environmental Sciences, University of New South Wales, Kensington, NSW, 2052, Australia; School of Life and Environmental Sciences, Deakin University, Burwood, Victoria 3125, Australia

**Keywords:** biodiversity conservation, environmental policy, extinction, invasive species, wildlife management

## Abstract

At least 40 Australian mammal spcies have been driven to extinction since European colonization in 1788. For conservation management to be effective, it is vital that the reasons for historical extinctions and ongoing declines are understood and remedied. A recent article (Wallach and Lundgren 2025) concluded that there was no compelling evidence that two introduced predators (domestic cats and red foxes) were primary causes of these mammal losses. We refute that article, finding substantial flaws in its premises, analyses, data, interpretations, and conclusions. Using multiple lines of evidence, we show that these two predators are strongly implicated in most Australian mammal extinctions and in the ongoing imperilment of numerous extant species. The devastating impact of cats and foxes on Australia’s mammals has been widely recognized by conservation managers who have, in response, implemented national programs to control these predators, producing widely recognized benefits for one of the world’s most remarkable native mammal faunas.

In essence, biodiversity conservation involves determining which factors are most detrimentally affecting a species or environment and then using this information to guide and enact policy and management responses. Elucidating the primary threat or threats is fundamental; if the threats are not identified, management efforts are likely to be misdirected, wasteful, and unsuccessful, and the affected species are likely to be extirpated.

In a recent article in this journal, Wallach and Lundgren (2025) reviewed evidence relating to the role of introduced predators (the feral domestic cat *Felis catus* and red fox *Vulpes vulpes*; hereafter, *cats* and *foxes*) in extinctions of endemic Australian terrestrial mammals since European colonization in 1788. The article concluded that there was no compelling evidence implicating cats and foxes in these extinctions. In the present article, we refute Wallach and Lundgren (2025), finding fault in its premises, analyses, data, interpretation, and conclusions. This dialogue is important because the factors that caused historic extinctions may also be causing the ongoing imperilment of related extant species; because the number of Australian mammal extinctions (40 species since 1788) is exceptional, vastly exceeding that for mammals in any other country over this period; and because introduced predators are also widely considered to be major factors in the extinction and imperilment of island faunas globally. We further note that this debate operates within three broader contexts: nativism, compassionate conservation, and invasive species denialism.

Before directly addressing the Wallach and Lundgren (2025) article, we briefly summarize some of the characteristics of the issue. Cats were introduced to Australia in 1788 and have since spread to occupy all habitats across the entire continent (Legge et al. 2017). Foxes established following introductions in the 1870s and have subsequently spread to occupy about 70% of the continent, with notable absences from the monsoonal north and the island of Tasmania (Tomlinson et al. 2025).

Reflecting millions of years of isolation, the Australian terrestrial mammal fauna is rich and highly distinctive, with approximately 340 mammal species at the time of European colonization and more than 90% endemism among the marsupial and rodent species (Baker et al. 2023). Since European colonization of Australia, at least 40 of these species (Burbidge 2023) have been driven to extinction (table 1). This constitutes more than 35% of the world’s 113 modern mammal extinctions (www.mammaldiversity.org). Many of these now-extinct Australian mammal species were formerly abundant and widespread. An additional 80 Australian mammal species are listed as threatened at national or global scales (supplement S2; Woinarski et al. 2014), around 40% have declined dramatically in distribution and population size (Fisher et al. 2003).

**Table 1.** List of Australian mammals classified as extinct since 1788.

Scientific name	Common name	Notes
*Dasycercus woolleyae*	Northern mulgara	Recently described species (Newman-Martin et al. 2023)
*Dasycercus archeri*	Southern mulgara	Recently described species (Newman-Martin et al. 2023)
*Dasycercus marlowi*	Little mulgara	Recently described species (Newman-Martin et al. 2023)
*Dasycercus cristicauda*	Crest-tailed mulgara	W&L. Recently redefined species (Newman-Martin et al. 2023)
*Thylacinus cynocephalus*	Thylacine	Extinction due to hunting or disease (Fisher and Burbidge 2025)
*Chaeropus ecaudatus*	Landwang, southern pig-footed bandicoot	W&L
*Chaeropus yirratji*	Yirratji, northern pig-footed bandicoot	
*Perameles eremiana*	Desert bandicoot	W&L
*Perameles fasciata*	Liverpool plains striped bandicoot	
*Perameles myosuros*	Marl	
*Perameles notina*	South-eastern striped bandicoot	
*Perameles papillon*	Nullarbor barred bandicoot	
*Macrotis leucura*	Yallara, lesser bilby	W&L
*Bettongia anhydra*	Desert bettong	W&L
*Bettongia haoucharae*	Little bettong	Recently described species (Newman-Martin et al. 2025)
*Bettongia penicillata*	Brush-tailed bettong	Recently redefined species (Newman-Martin et al. 2025)
*Bettongia pusilla*	Nullarbor dwarf bettong	
*Caloprymnus campestris*	Desert rat-kangaroo	W&L
*Potorous platyops*	Broad-faced potoroo	W&L
*Lagorchestes asomatus*	Kuluwarri, central hare-wallaby	W&L
*Lagorchestes leporides*	Eastern hare-wallaby	
*Notamacropus greyi*	Toolache wallaby	extinction likely due to habitat modification and hunting
*Onychogalea lunata*	Crescent nailtail wallaby	W&L
*Crocidura trichura*	Christmas Island shrew	Likely recent extinction (Woinarski et al. 2023b), due to introduction of *Rattus rattus*, disease, and wolf snake *Lycodon capucinus*
*Pteropus brunneus*	Dusky flying-fox	Cause of extinction is obscure
*Nyctophilus howensis*	Lord Howe long-eared bat	Extinction likely to have been due to introduced predator (*Rattus rattus*)
*Pipistrellus murrayi*	Christmas Island pipistrelle	Extinction since 2000. Extinction due to introduced predator (wolf snake; Woinarski 2018)
*Conilurus albipes*	White-footed rabbit-rat	W&L
*Conilurus capricornensis*	Capricorn rabbit-rat	W&L. Only reported from subfossils, with some probably postdating 1788
*Leporillus apicalis*	Lesser stick-nest rat	W&L
*Notomys amplus*	Short-tailed hopping-mouse	W&L
*Notomys longicaudatus*	Long-tailed hopping-mouse	W&L
*Notomys macrotis*	Large-eared hopping-mouse	W&L
*Notomys mordax*	Darling Downs hopping-mouse	W&L
*Notomys robustus*	Broad-cheeked hopping-mouse	
*Pseudomys auritus*	Long-eared mouse	W&L
*Pseudomys glaucus*	Blue-grey mouse	W&L
*Melomys rubicola*	Bramble Cay melomys	Extinction since 2000, due to climate change and sea water inundation
*Rattus macleari*	Maclear’s rat	Extinction due to introduced *Rattus rattus* and disease
*Rattus nativitatis*	Bulldog rat	Extinction due to introduced *Rattus rattus* and disease

*Note:* Extinctions clearly attributable to factors other than cats or foxes are indicated in the Notes column. Species included in the analyses in Wallach and Lundgren (2025) article are signified W&L.

Even for recent and relatively well documented losses, it may be challenging to identify a cause of extinction or imperilment because multiple factors may play a role, to varying extents and in variable orders (Emery et al. 2021). The evidence base is extremely limited for some extinct Australian mammals. A few Australian mammal species were never documented by Western scientists while they persisted (Cramb and Hocknull 2010), others are known from only a single specimen (Burbidge et al. 1988), and many disappeared over 100 years ago, with no written description of their ecology (Fisher and Blomberg 2011).

## Critique of Wallach and Lundgren (2025)

Wallach and Lundgren (2025) asserted that evidence implicating introduced foxes and cats in the Australian mammal extinctions is insufficient to demonstrate causality. Their argument has three main premises. Premise 1 is that if cats and foxes were responsible for these extinctions, then the last record of a nationally or regionally extinct native mammal species from a region must postdate the arrival of cats and foxes to that region. Their review also sought to infer the causes of historic extinctions by assessing the response of *extant* Australian mammals to introduced predators. Specifically, they tested (premise 2) that native mammal abundance should increase when management reduces the abundance of introduced predators and (premise 3) that, across space or time, native mammal abundance is negatively correlated with introduced predator abundance at local or regional scales. In the present article, we scrutinize the assumptions and evidence presented in Wallach and Lundgren (2025). Our conclusions are also summarized in table 2.

**Table 2.** Summary of claims and flaws in Wallach and Lundgren (2025; *W&L* below) claiming insufficient evidence to implicate cats and foxes in losses of Australian mammal fauna.

Premise	W&L claim	Premise or analysis are flawed because:
1. Extinctions of a native mammal species in an area must postdate the arrival of cats or foxes in that area	Dates of extinctions mostly occur before cats or foxes arrive	Premise correct, but analysis flawed because: The last record of a native mammal species is an unreliable marker of its extinction date, given the paucity of observations and surveys in arid and semiarid Australia, especially in the early decades after European colonisation There are many errors in W&L’s compilation of the last records, including last record dates that are incorrect, and record duplicates (see supplement S1). The compilation of last records includes repeated nonindependent observations for one species (30% of all records) that is known to coexist with cats in some circumstances. Eye-witness accounts of population extirpation following the arrival of cats or foxes by First Nations People and early European naturalists were ignored. The extinction process is affected by variability in the environment, and interactions between cats and foxes. Cats arrived at locations earlier than foxes so any extinctions due to cats could have pre-dated fox arrival but this is not evidence of no fox impacts. Despite all the flaws noted above, reanalysis of the data in W&L clearly shows that all population extirpations happened after cat arrival (figs. 1 and 2).
2. Cat or fox management programs should reduce predator numbers and increase abundance of predator-affected native mammals	Management of foxes results in increases for native mammals; no correlation for cats	Premise muddles two components: whether management reduces cat or fox density, and whether there is a relationship between cat or fox density and native mammal abundance (premise 3). A more appropriate premise would be: “When cat or fox density is *effectively and sufficiently* reduced by management, native mammals susceptible to those predators should increase.” Analysis flawed because it: Lumped effective with ineffective cat or fox management, and effective with ineffective monitoring. Ignored the substantial evidence from (less gradational and more robust) translocation programs, showing that when management removes, excludes, or strongly reduces cats and foxes at a site, translocations of cat- and fox-susceptible mammals are likely to succeed; translocations to sites without such cat or fox management fail.
3. The abundance of native mammals should be negatively correlated (over space or time) with the abundance of invasive predators	There are no such correlations	Premise flawed because there is no reason to expect a negative relationship between cats or foxes and native mammals: There are many situations where a POSITIVE relationship between cats and foxes and native mammals is expected, including ○ In arid and semiarid Australia, where the abundance of both cats or foxes and native mammals increases after rain. ○ Cats or foxes and native mammals may all be more abundant in higher productivity than lower productivity sites. ○ Cat or fox abundance may be low in an area from which they have consumed almost all native mammals. Where a negative relationship between cats or foxes and native prey exists, it may be hard to detect because: ○ The effect of cats or foxes on some native mammal species is so strong that they almost never coexist. ○ The most susceptible mammals are already extinct (or locally extirpated) and are not available to respond to predator control (extinction filter). The population response of native mammals to cat or fox predation depends on which predator is present, what alternative prey is present, and the habitat structure and condition of ecosystems; therefore, consistent correlations across all species are not expected. Analysis flawed because W&L included only 19 studies, some notable omissions include: ○ Substantial expansions in the distributions of cat- and fox-susceptible mammals in southern Australia following reductions in cat and fox abundance, caused by biocontrol of rabbits. ○ Multiple examples of native mammal populations that persisted on cat- and fox-free islands but were extirpated from their mainland range following the spread of cats and foxes.

### Premise 1: Extinction must post-date predator arrival

Wallach and Lundgren's (2025) first premise is that extinctions of a native mammal species in an area must postdate the arrival of cats or foxes in that area. Wallach and Lundgren (2025) collated the dates of last records for 178 cases of local extirpation or extinction and matched those dates with estimates of the dates of arrival at the site of cats and of foxes. From analysis of this database, they reported that many mammal populations were extirpated before the arrival of cats or foxes, and therefore, those predators could not be the cause of those extinctions. To come to this conclusion, they assumed that the last documented record of a species was the extinction date. Given that most extinct Australian mammal species occurred in remote areas seldom visited by explorers or museum collectors, the date of last collection or other documented record is likely to be an extremely unreliable marker of extinction date (Fisher and Blomberg 2011).

Similarly, the date of first arrival of cats and foxes is also difficult to determine because a species can be present at low density and remain undetected. Cats and foxes are therefore likely to have been present in an area before their first documented detections, particularly in remote areas sparsely populated by humans.

A likely more accurate source of extinction dates comes from eye-witness accounts of First Nations peoples who lived in these remote areas and had intimate knowledge of their land and of the species present. There has been much documentation of this evidence for the timing of extirpation of many native mammal species (Johnson and Roff 1982, Burbidge et al. 1988, Burbidge and Pearson 1989, Tunbridge 1991, Ziembicki et al. 2013), typically demonstrating that many now-extinct mammals persisted long after the dates inferred from other sources. Only 9 of the 178 (approximately 5%) last dates collated by Wallach and Lundgren (2025) were from such First Nations sources. The neglect of such documented Indigenous knowledge in Wallach and Lundgren (2025) is an important oversight.

Nonetheless, some extirpations of Australian mammals were closely witnessed by Western scientists (e.g., Wood Hoy 1923, Le Souef 1923, Finlayson 1961, Jones 1968). For example, the museum collector Charles Hoy wrote in the 1920s shortly after the spread of fox to Eyre Peninsula, South Australia, “the poor luck in mammals is explained by the total extermination of most of them. This has been caused mainly through the introduction of foxes and cats… The fox has only been plentiful during the last three and four years… [and] native mammals have been completely wiped out during the last few years.” Likewise, at Tamworth, in northern New South Wales, in 1919, Hoy wrote “even as late as three years ago, the district was literally overrun by [wallaby species] but soon after the advent of the foxes they disappeared so that now *Onychogalea frenata* and *Aepyprymnus rufescens* are extinct and the others rapidly approaching extinction. *Dasyurus* was wiped out about eight years ago by the domestic ‘tabby' cat in the wild" (Short and Calaby 2001).

**Figure 1.**
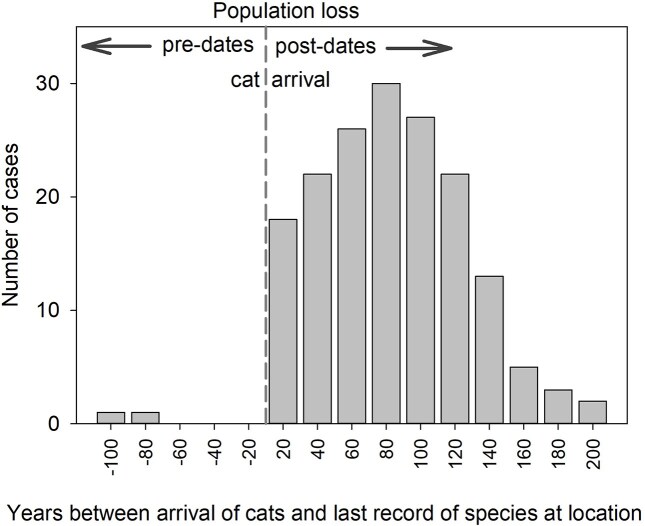
Timing of extirpation of a native mammal population from a location relative to the time of arrival of feral cats at that location. Data are taken directly from Supplement 1 in Wallach and Lundgren (2025), using the last record of the native species, and the earliest arrival date from the range of arrival dates of cats to that location. Based on this information, extirpation of a native mammal occurs before cats arrive in only two cases, and these are where Wallach and Lundgren (2025) arbitrarily give the last record dates of 1788 and 1789 (i.e., time of European colonization, in 1788) for undated subfossil material. Of the 178 cases given in the Supplement, eight are omitted from this graph because: they were repeats of another row (n=4), there is no date for the last record of the species (n=1), the species is still present at the location (n=2), or the species never occurred at the location (n=1). Figure prepared by authors..

Even with such shortcomings in assumptions, the data compiled by Wallach and Lundgren (2025) for timing of extirpation do, in fact, show strong support for cats (at least) as a factor contributing to these extinctions. We graph their data in figure 1, illustrating that all valid last records of the extirpations reported in Wallach and Lundgren (2025) occurred *after* the arrival of cats.

**Figure 2.**
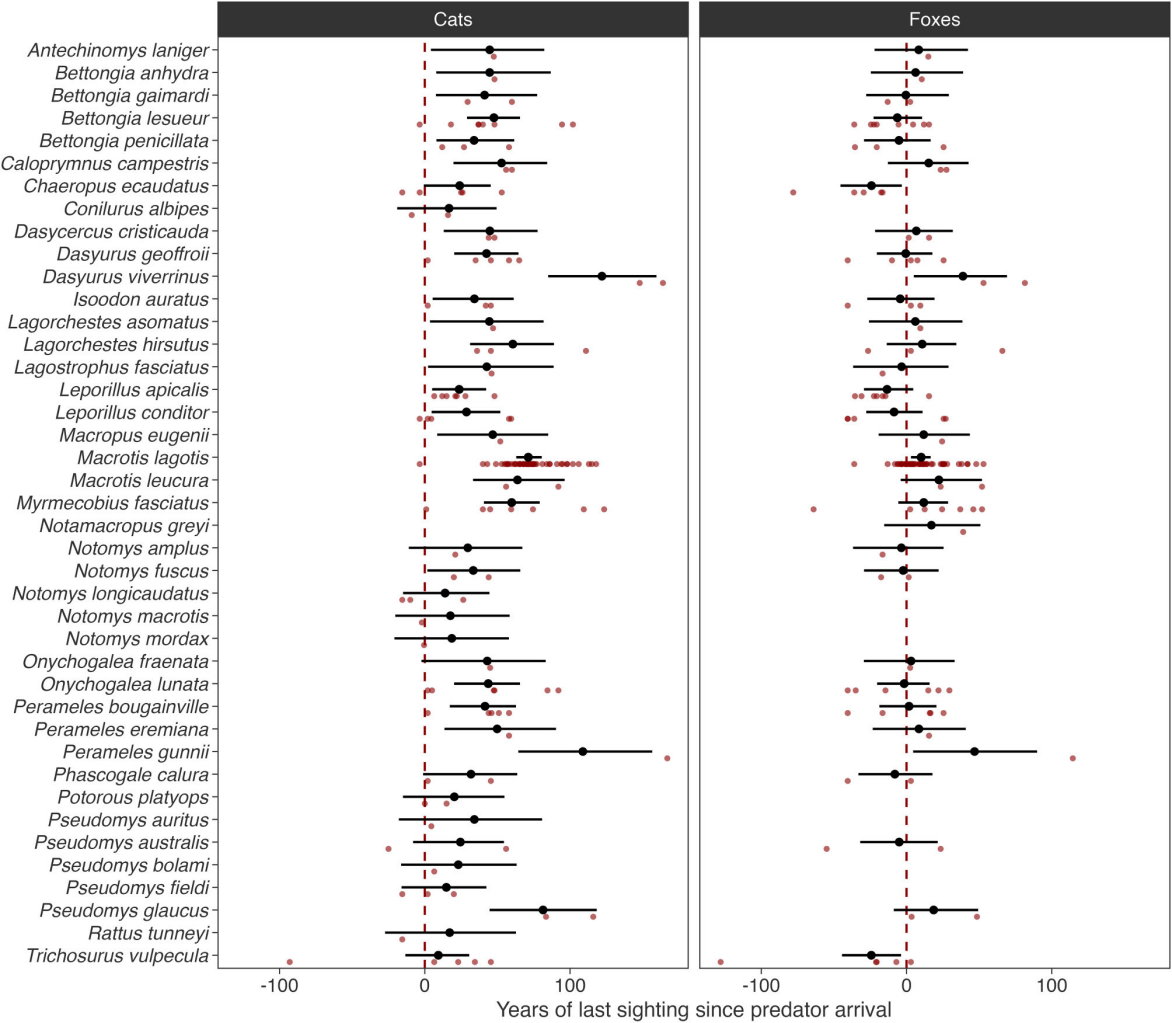
Timing of extirpation of native mammal populations at a location relative to the arrival of cats and foxes at that location. Estimates (posterior medians and 95% highest density intervals [HDIs]) show prey-level random intercepts for each predator from an interval-censored model accounting for uncertainty in the response variable. Predator averages were 41.1 (95% HDI: 34.9, 47.7) years for cats and 4.1 (95% HDI: -2.5, 11.4) for foxes. Red points (raw data) are the empirical means of the minimum and maximum possible years since the last sighting. Figure prepared by authors.

We also reanalyzed the data given in Wallach and Lundgren (2025) by statistically modeling their last records since predator arrival (separately for cats and foxes) with mammal species-level random effects, using interval censoring to capture the provided uncertainty in the response data, in the Stan program (Carpenter et al. 2017). We filtered out records listed as uncertain by Wallach and Lundgren (2025) and additionally censored some erroneous records (see supplemental file S1). This model estimated the number of years after predator arrival that each species was last recorded: This averaged 41 years for cats and 4 years for foxes (figure 2). For *all* extinct mammal species in the Wallach and Lundgren (2025) database, the posterior median of year of last sighting was *after* cats' arrival. No comparable pattern of loss was evident for foxes, likely either because the earlier arrival of cats had already caused extirpation or because foxes drove susceptible mammals extinct so rapidly that the confidence intervals overlap the last sighting dates (e.g., Short 1998 showed that bettongs went extinct within 5 years of the fox's arrival in each district of New South Wales). The period that these native mammals were able to persist with foxes and cats would have differed among species because of selective predation, variable life history and ecological traits, and differences among species and locations in the availability of ecological refuge sites (sites less suitable for cats or foxes or offering more shelter for vulnerable native mammals; Geyle et al. 2025).

Furthermore, the analysis by Wallach and Lundgren (2025) treated cats and foxes as independent threats, but the combined impact of these two predators is relevant in at least some cases (e.g., Lundie-Jenkins et al. 1993, Marlow et al. 2015). This issue is particularly important in their analyses because 53 of the 178 cases (30%) considered by Wallach and Lundgren (2025) were instances of local extirpation of a single extant species, the greater bilby *Macrotis lagotis*. The bilby can coexist with low densities of cats (Moseby et al. 2019, Chen et al. 2024), and the combined effects of threatening processes are likely important for this species rather than simply the arrival date of the cat or the fox. We note also that the high proportion of bilby cases in the analysis of Wallach and Lundgren (2025) introduces issues of nonindependence and undue influence of a single species to the analysis, which was appropriately handled by our statistical analysis.

Wallach and Lundgren (2025) presented data relating to the timing of extinction for only 18 of the 40 extinct Australian mammals but amplified their database by also considering regional losses of 23 extant species. We recognize that 9 of the 40 Australian mammal extinctions are clearly attributable to factors other than cats and foxes (table 1): These are appropriately excluded in Wallach and Lundgren's (2025) analysis. However, many of the extinctions of other species not considered in Wallach and Lundgren (2025) are clearly attributable to cats or foxes. For example, Newman-Martin and colleagues (2025) reported that *Bettongia haoucharae* “went extinct sometime during the 1920s due to the spread of foxes in this region. This is a similar extinction window to other small mammalian fauna in the region, such as *Perameles papillon*… and *Dasycercus archeri*,… which were both last seen alive on the Nullarbor in the 1920s.”

### Premise 2: Management of introduced predators will increase abundance of native mammals

Wallach and Lundgren's (2025) second premise is that management programs aimed at controlling introduced predators should result in reductions in the numbers of those predators and concomitant increases in the abundance of predator-affected native mammals. Wallach and Lundgren (2025) collated data from some predator control programs and reported that there was indeed such a correlation for foxes but not for cats. However, these data are complex to interpret, because the responses of cats (particularly) and foxes to control programs are variable, may interact, may be transient, and are often highly localized (Moseby and Hill 2011, DCCEEW 2024) and because many other factors may also influence the abundance of native species. This is especially true for cats, for which landscape-scale control is very difficult, rarely effective in the long term, and highly dependent on seasonal conditions and alternative food availability (Christensen et al. 2013, Moseby et al. 2021, DCCEEW 2024).

The cat and fox abundance data considered in Wallach and Lundgren (2025) are gradational and ephemeral. A far more definitive and robust contrast (with cats and foxes being either absent or present) comes from studies and conservation programs that eradicate and then exclude these introduced predators from an area. Many such studies have reported success for imperiled Australian native mammals reintroduced into predator-proof fenced exclosures (e.g., Woinarski et al. 2023a, Moseby et al. 2011, Kanowski et al. 2018, Legge et al. 2018), and the failure of other reintroductions into environments with cats or foxes (e.g., Christensen and Burrows 1994, Short 2016). Wallach and Lundgren (2025) discounted such evidence on the perplexing grounds that ‘‘*… some have pointed out that predation by foxes and cats can cause reintroduction failures. Although this observation is relevant to the practice of reintroductions, it is not relevant to the question of why extinctions occur*.” We disagree. It is only through removing putatively causal factors and using replicated experimental trials that the possible cause of population declines toward extinction can be tested (Caughley and Gunn 1995, Côté et al. 2016).

### Premise 3: The abundance of introduced predators and of native mammls should be inversely correlated

If predators were implicated in the extinctions, as is argued in Wallach and Lundgren's (2025) third premise, then the abundance of extant native mammals in a landscape should be negatively correlated (over space or time) with the abundance of the nonnative predators. This premise is flawed for many reasons. In some circumstances, native mammal species that are susceptible to cat or fox predation may be able to withstand higher densities of cats and foxes. For example, if resources are abundant, then the vital rates of susceptible species may be sufficient to withstand high (or higher) levels of cat or fox abundance and predation. Likewise, if there is sufficient shelter to reduce predation, then susceptible species may be able to persist at high (or higher) densities despite the presence of cats and foxes (Moore et al. 2019). Furthermore, at least in arid and semiarid Australia, the abundance of both introduced predators and native mammals fluctuates strongly and synchronously with rainfall events (Read and Bowen 2001, Letnic and Dickman 2006, Greenville et al. 2014), leading to a positive correlation over time in abundance of predators and prey species. Spatially, across a range of local environments, both cats and foxes and native mammals may be more abundant in higher productivity sites, leading to a positive correlation. Cats and foxes can also be attracted to and reach high abundance at sites where native mammal prey are most abundant, leading again to a positive correlation (Miritis et al. 2020, Geary et al. 2022). Cat or fox abundance may be low in an area from which they have consumed almost all native mammals, leading to no correlation or a positive correlation. Furthermore, even single cats or foxes can have devastating impacts on native mammal fauna (Moseby et al. 2015), so linear associations in predator and prey abundance and impacts should not always be expected (Sheldon et al. 2023). There is also an extinction filter effect (Balmford 1996), where the most susceptible mammals are already extinct (or extirpated) and not available to respond to predator control. Furthermore, the susceptibility of mammals to predation is variable depending on which predators and prey are present and the habitat structure and on the conditions within the ecosystems (Radford et al. 2018, Miritis et al. 2020), so consistent correlations across all species are not expected.

In summary, the perspective presented in Wallach and Lundgren (2025) that introduced predators did not cause the extinction and ongoing decline of Australian mammals is not supported by the evidence (table 2) and has little or no credibility among those engaged in conservation management in Australia. Furthermore, they do not offer a plausible or more convincing alternative hypothesis for Australia’s globally extraordinary record of recent mammal extinctions.

## Additional evidence not considered in Wallach and Lundgren (2025)

**Figure 3.**
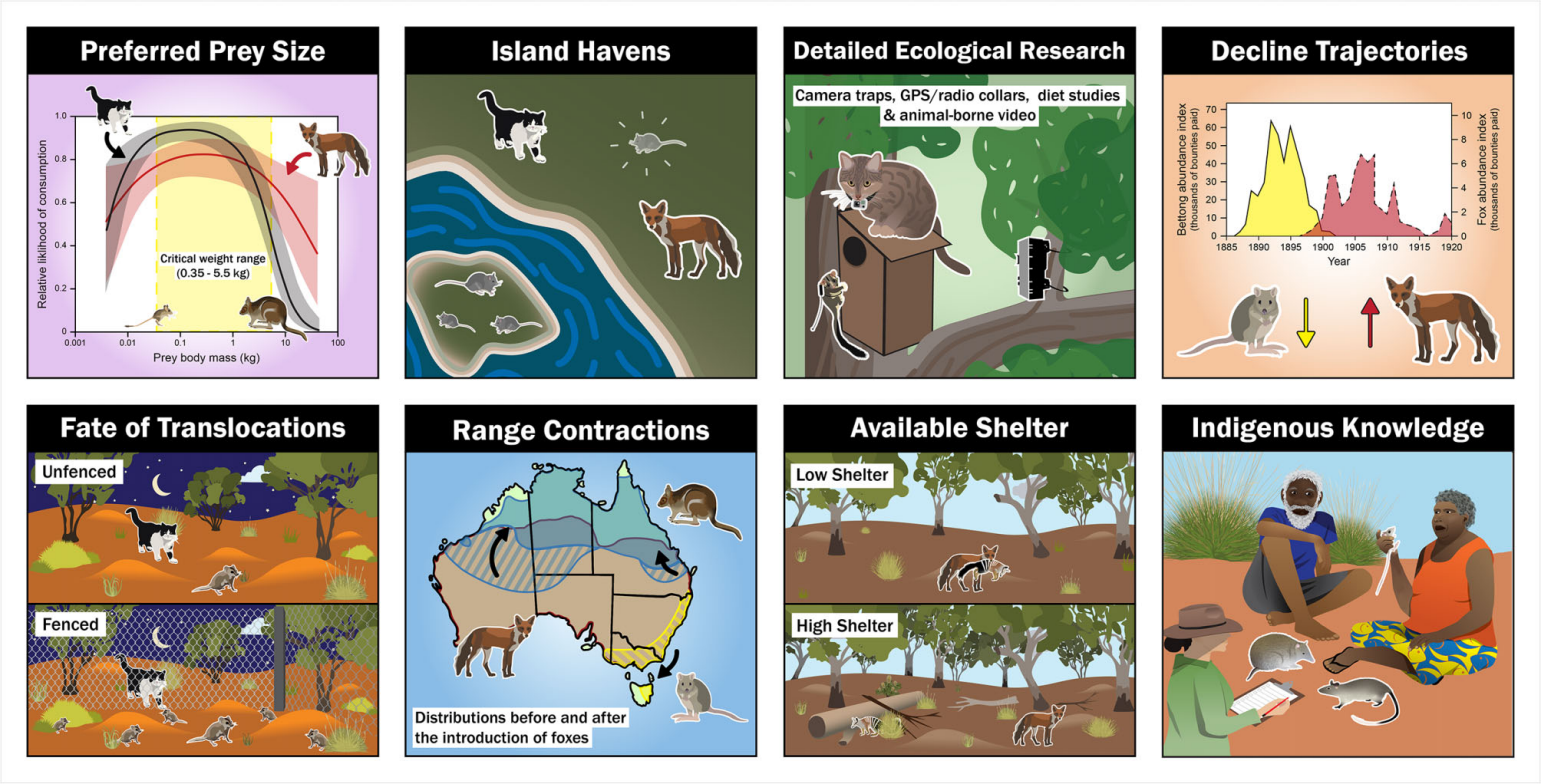
Graphic of main lines of evidence implicating introduced predators (cats and foxes) in losses of Australian native mammals. Figure prepared by authors.

In figure 3, we portray the major components of the evidence base implicating cats and foxes in extinctions and ongoing declines of Australian mammals. A compelling strand of evidence is the recurring pattern for many Australian mammal species of extirpation over vast mainland ranges, but persistence of populations on islands to which cats or foxes were not introduced (Burbidge 1999). The inference that can be drawn from these patterns are especially strong for pairs of congeneric species for which both species have been extirpated from their entire mainland ranges (which were uniformly invaded by cats and foxes but over which the occurrence of other putative threats was highly variable) but for which one of the pair of species has persisted because it had populations on islands where cats and foxes were not introduced, whereas the other did not have such island populations. These paired cases include the extant greater stick-nest rat *Leporillus conditor* (which has survived because it had a population on Franklin Islands) and the lesser stick-nest rat *Leporillus apicalis* (extinct, no island populations), the burrowing bettong *Bettongia lesueur* (Barrow, Bernier, Boodie, Dorre islands) and the desert bettong *Bettongia anhydra* (extinct), the Shark Bay bandicoot *Perameles bougainville* (Bernier, Dorre islands) and the desert bandicoot *Perameles eremiana* (extinct), and the rufous hare-wallaby *Lagorchestes hirsutus* (Bernier, Dorre islands) and the central hare-wallaby *Lagorchestes asomatus* (extinct; Burbidge et al. 1997).

Furthermore, some island populations were also extirpated following the introduction of cats or foxes. One such case, *Bettongia penicillata francisca*—restricted to a single island—was well documented: “One animal, the quite recent extermination of which we must greatly regret, is the small unknown creature which used to live in great numbers on St. Francis Island. Mr. Lloyd… described them as very small wallabies, creatures which used to hop into the homestead and eat scraps thrown to them from the table. Cats were liberated in order to destroy it, and they have done their work with thoroughness” (Wood Jones 1922).

Conservation translocations of imperiled Australian mammals mostly succeed when cats and foxes are absent or heavily controlled but almost always fail when cats and foxes are not sufficiently controlled. Cats and foxes have been eradicated from an expanding haven network of islands and mainland fenced areas (exclosures) around Australia (Legge et al. 2018). Numerous imperiled native mammals have then been translocated to these havens. In some cases, these translocations are effectively before–after–control–impact experimental designs, whereby the native mammals that were eliminated (by predators) prior to predator exclusion, have subsequently flourished in havens after predators were excluded (impact) but failed to establish in comparable habitat when translocated outside the exclosure where predators remain (control; Moseby et al. 2011). Reintroductions have been successful outside fenced areas only where introduced predators have been intensively and effectively controlled (Moseby et al. 2021) or, for some species that are more susceptible to foxes than cats, where sites have low cat density but no foxes (e.g., eastern barred bandicoot *Perameles gunnii* introductions to Victorian islands; Parrott et al. 2017). In many cases where control programs have not reduced the densities of introduced predators sufficiently, the reintroductions have failed because of predation on the released animals (Moseby et al. 2011, Bannister et al. 2016, Hardman et al. 2016, Short 2016, Robinson et al. 2020).

**Figure 4.**
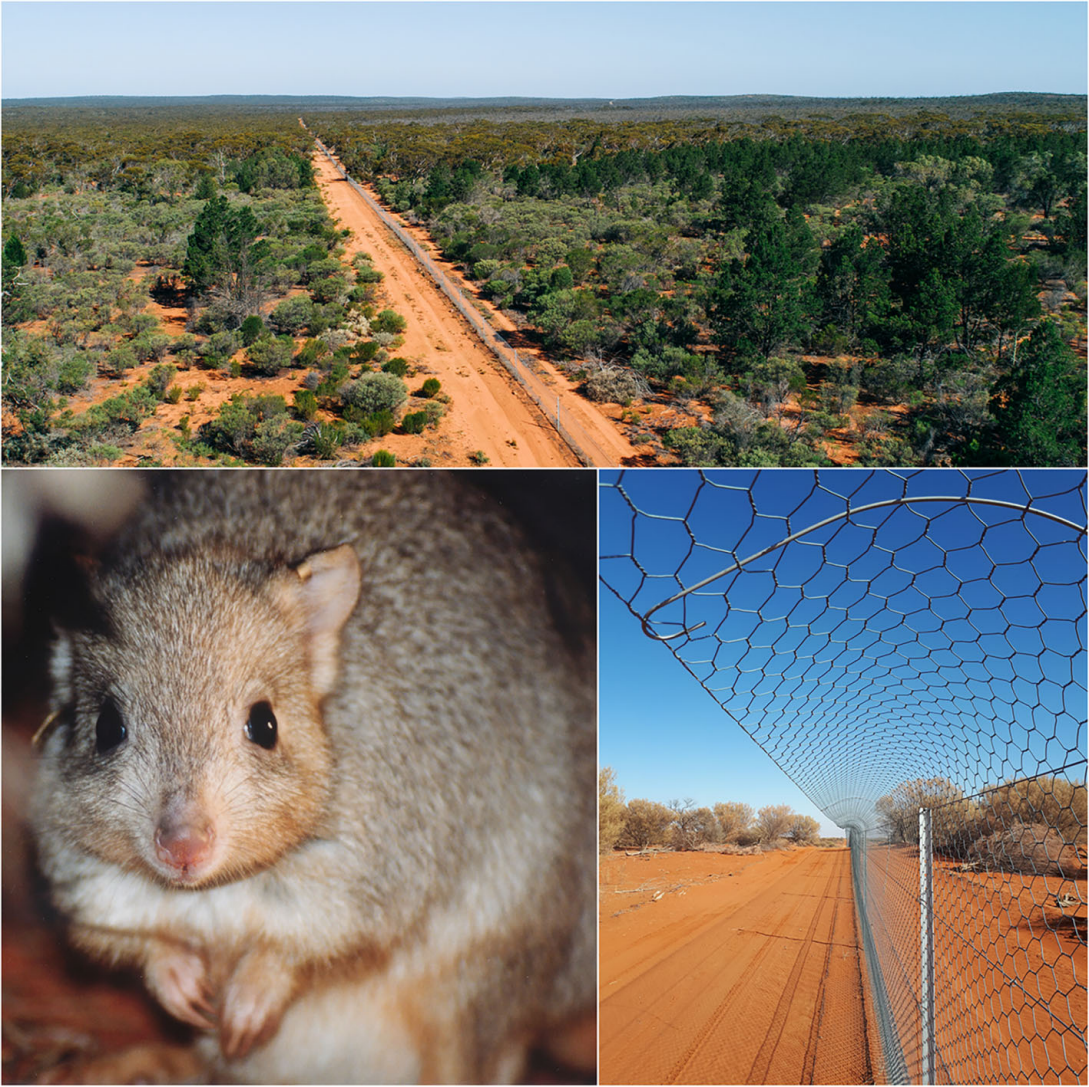
An example of an endemic mammal species, the burrowing bettong (Bettongia lesueur) extirpated from its formerly vast mainland range and now present on the mainland only as populations reintroduced to fenced areas that exclude cats and foxes. Photo credits: top (Robert Lang); bottom left (Andrew Freeman); bottom right (K. Moseby).

The success of translocations—predicated on recognizing the significant threat of cats and foxes and, therefore, the need to control them—is well illustrated by the case of the burrowing bettong (figure 4). This species formerly occurred across more than 1 million square kilometers (km^2^) of mainland Australia and on four islands but was extirpated from its entire mainland range (Short and Turner 1993). Reintroductions from islands have returned the species to parts of its former mainland range but have succeeded only in fenced areas from which cats and foxes have been eradicated (Finlayson et al. 2010, Moseby et al. 2011) or where foxes have been eradicated and cats heavily suppressed. Instances of surplus killings by foxes that breached some fenced areas resulted in rapid and large population losses of bettongs (36%–77%), even when rabbits were 700 times more abundant than bettongs (Short et al. 2002).

Cats and foxes have exerted and continue to exert an enormous toll on Australian mammals, and that toll is concentrated on a subset of species. Cats kill an estimated 452 million Australian native mammals per year, and foxes kill an estimated 104 million per year (Stobo-Wilson et al. 2021). This toll falls inequitably across mammal species, because the ecological traits and life history characteristics of some native species render them more susceptible to such predation pressure and because of prey selectivity by cats and foxes (Read et al. 2019b, 2024, McGregor et al. 2025).

In addition to predation, cats can cause the death of mammals through pathogens and a range of protozoan sarcocystid parasites; most notably, cats are the only definitive hosts of the protozoan parasite *Toxoplasma gondii*, and, therefore, this parasite would not occur in Australia were it not for the introduction of cats. Many Australian mammal species are now affected by toxoplasmosis, which is a significant cause of mortality or morbidity (especially central nervous system abnormalities; Canfield et al. 1990, Parameswaran et al. 2010, Groenewegen et al. 2017, Lynch et al. 2025). These lethal or sublethal effects further compound the direct predatory impacts of cats.

In contrast to the assertions presented in Wallach and Lundgren (2025), populations of many declining mammal species respond positively to intensive reduction or exclusion of cats and foxes. Early work documented the spectacular recovery of mammals after poison baiting for foxes (e.g., Kinnear et al. 1988, 2002, Burrows and Christensen 2002), leading to the implementation of long-term and large-scale control programs for cats and foxes initially in Western Australia (Possingham et al. 2004, Department of Parks and Wildlife 2017). Subsequent comparable large-scale and ongoing fox baiting programs to protect biodiversity are now in place and are achieving conservation outcomes in many other Australian regions (Dexter et al. 2007, Dexter and Murray 2009, Brandle et al. 2018, Robley et al. 2023). Many control programs for foxes or cats have led to increases in the abundance and distribution of many threatened mammals (Hunter et al. 2018), including quolls *Dasyurus* spp. (Palmer et al. 2021), black-flanked rock wallabies *Petrogale lateralis* (Read et al. 2025), and yellow-footed rock-wallabies *Petrogale xanthopus* (Sharp et al. 2014). Indirect reductions in cats and foxes have also led to increases in populations of threatened mammals. For example, the accidental release of rabbit hemorrhagic disease virus caused a sudden and drastic reduction in Australian rabbit populations in 1995, leading to a decline in cats and foxes, which was then followed by a significant increase in the distribution and abundance of some threatened Australian desert mammals (Pedler et al. 2016).

Collectively, the available evidence strongly implicates cats and foxes as the primary threats to extant imperiled Australian mammals and the major causes of the loss of extinct species (table 2). This contrasts markedly with the situation in many other continental landmasses, where habitat destruction and modification and hunting are the main causes of most mammalian extinctions (Schipper et al. 2008). Of course, we recognize that other factors have contributed to some declines and extinctions of Australian mammals (Johnson 2006, Kearney et al. 2023). Disruption or interruption of precolonial Indigenous land management (Bird et al. 2018), land clearance for agriculture, urbanization and mining, inappropriate fire regimes (Kearney et al. 2023), overgrazing by the introduced rabbits *Oryctolagus cuniculus* and domestic livestock, disease, the loss and ongoing persecution of dingoes and their ecological functions (Letnic et al. 2011), and climate change (Wagner et al. 2020) have all played a part in the decline and extinction of some Australian land mammals (Johnson 2006) and have likely facilitated or exacerbated predation impacts by cats and foxes (Doherty et al. 2015). We do not claim that control of cats and foxes will fix all these problems.

## Implications for conservation and the future

In response to the historical losses and the continuing decline and perilous state of Australia’s native mammal fauna, there have been decades-long, evidence-based, and collaborative efforts to control cats and foxes at sites across the continent. The extent and success of many of these efforts are exceptional at a global scale.

One major ingredient has been the formal recognition of feral cats and foxes as key threatening processes to Australian biodiversity under national environmental legislation. This recognition has led to threat abatement plans, first established for both feral cats and foxes in 1999 (Environment Australia 1999) and continued to the present (DCCEEW 2024, 2025). These threat abatement plans collate the significant scientific evidence for impacts, serve to coordinate and integrate the management of feral cats and foxes across all Australian states and territories, provide a legal foundation for actions including an obligation to control these threats on lands owned by the Australian government, help coordinate information, and set targets to benefit the most imperiled and predator-susceptible native species and the places where they occur. Policy relating to and management of cats and foxes have also advanced in individual Australian states and territories.

The importance of cat- and fox-free islands for the conservation of Australian biodiversity has been long recognized, and predator-susceptible threatened mammals have been introduced to many such islands from at least the 1970s (Legge et al. 2018). Recognizing that eradication of cats and foxes from the Australian mainland is unfeasible currently, but that eradication is tractable on some islands, a long-standing effort has resulted in the eradication of feral cats from 31 islands totaling 967 km^2^ (the largest being Dirk Hartog Island, 633 km^2^; Algar et al. 2020) and foxes from 17 islands totaling 185 km^2^ (the largest being Phillip Island, 101 km^2^). Predator-susceptible native mammals have now been translocated to many of the islands from which introduced predators have been eradicated; almost all of these translocated populations are now flourishing (e.g., Cowen et al. 2023). The importance of islands has also been recognized proactively in ongoing biosecurity settings that aim to prevent the introduction and establishment of threatening nonnative predators (and other nonnative species) to islands of biodiversity significance (DCCEEW 2024).

Complementing this predator-free island network, cats and foxes have also been eradicated from 40 fenced sites on mainland Australia (with a total area of 826 km^2^) since the 1980s. Threatened predator-susceptible native mammals have since been translocated to these sites, where they survive without supplementary food. In many of these exclosures, the remnant *in situ* populations of native mammals have also increased (e.g., Moseby et al. 2025). Nongovernment conservation organizations have been and continue to be instrumental in the establishment and maintenance of this haven network (Moseby et al. 2009, Kanowski et al. 2018), complementing the contributions of government agencies.

Collectively, these islands and mainland exclosures constitute a network of 140 havens, with a total area of 3761 km^2^, and they now support 250 populations of 45 native mammal taxa (27 species) that are highly or extremely susceptible to cats or foxes. However, although this expansion has done much to reduce the risk of extinctions and to increase the population size of many highly imperiled Australian mammals, it collectively represents a minute proportion of the former range of many of these mammal species. Cats and foxes still occur on approximately 98 and 53 Australian islands, respectively. Reestablishing threatened mammal species in larger areas of their former ranges will rely on effective management of cats and foxes to densities that facilitate coexistence at landscape scales, combined with habitat recovery and opportunities for native species to adapt to these nonnative predators (Moseby et al. 2019).

Practitioner expertise and innovation have been important to the management of cats and foxes in Australia. Technical advances have resulted in an increase in the uptake of baits selectively by target species (cats and foxes), novel methodologies that improve baiting effectiveness, and increases in the humaneness of baits (e.g., Johnston et al. 2020). Recognizing typically lower uptake of baits by cats than by foxes, novel baits such as Eradicat have been developed, as has a poison-delivery device that can specifically recognize cats and then propel poison onto their body, taking advantage of the cat’s propensity for grooming to ingest the poison (Read et al. 2019a, Moseby et al. 2020).

Furthermore, increasing evidence demonstrates the importance of managing fire or large herbivore populations and grazing pressure for maintaining ground-level plant cover and complexity, which may allow susceptible native mammals to persist in the presence of introduced predators (Legge et al. 2011, 2019, Hradsky 2020, Doherty et al. 2022, Neave et al. 2024).

Management of introduced predators has been remarkably successful for the conservation of Australia’s remaining extant mammals. On the Australian mainland, the spate of mammal extinctions has (so far) ended, with no extinctions since the 1960s (although there have been three extinctions of island endemic mammals since 2000; Woinarski et al. 2023b, 2025). Because of translocations and havens, the population sizes of Australia’s most imperiled mammal species have increased by an average of 680% over the period 2000 to 2017 at sites where invasive predators are effectively controlled, whereas mammal populations exposed to introduced predators continue to decline (by an average of 80% over that same period; Tulloch et al. 2023). Although most threatened mammals are still a long way short of full recovery (Read et al. 2023), and many are still in decline, at least 16 species are now increasing in population size and distribution (Woinarski et al. 2023a). A spectacular example of such success is that of the eastern barred bandicoot: Once extirpated on mainland Australia (i.e., outside of Tasmania), it now thrives on three Victorian islands, including Phillip Island, from which foxes were eradicated by 2017 (Adriaanse et al. 2024).

## Contested perspectives

Dispute about the role of invasive predators in the extinction and imperilment of Australian mammals sits within a broader debate (Haubrock et al. 2025). Some have argued that there is no ethical base for nativism—the preferencing of native species ahead of species whose origins lie elsewhere biogeographically (Peretti 1998). We consider this an absurd and neocolonialist proposition in an Australian context, where the native biota represents an extraordinary and highly distinctive legacy of millions of years of isolation, is of great cultural and spiritual significance for First Nations peoples who acknowledge a multigenerational and pervasive existential obligation to care for their country and its biota, and is considered by many Australians, Indigenous and non-Indigenous alike, as a key ingredient of national identity (Biodiversity Council 2025).

A related perspective is invasive species denialism (e.g., Russell and Blackburn 2017). This perspective proposes that the impacts of nonnative species are overstated, that attempts to control or eradicate them will almost always be futile, and that nonnative species are now an intrinsic component of novel ecosystems that are or will be the inevitable consequence of the way in which humans have altered the planet (see Ricciardi and Ryan 2018). We refute this too: There is incontrovertible evidence of the severe detrimental impacts of cats and foxes on Australian mammals, of the potential for those impacts to be mitigated, and of the recovery of many native species where these invasive predators are effectively controlled.

Another contextual perspective is compassionate conservation, which claims to prioritize the welfare and rights of individual animals even if at the expense of populations, species, or ecosystem conservation (Wallach et al. 2015, Wallach et al. 2020). At its core, this approach opposes the killing of any animal by humans. As a construct, it only works for invasive species management if the indirect welfare impacts (e.g., predation) on co-occurring animals in the recipient ecosystem are discounted. Although conservation should always seek to minimize negative impacts on animal welfare, we cannot avoid making tough choices to control—in the most humane and effective way possible—problematic nonnative species in order to conserve threatened native species and the ecosystems they shape. This choice is supported by most Australians (Zander et al. 2021). Framing the conservation practitioner’s choice as either killing a nonnative predator or avoiding any deaths at all is misleading. The real choice is between directly killing a nonnative predator and sentencing many more native animals to die because the nonnative predators are left alive.

## Conclusions

We welcome objective and reasonable scientific scrutiny of long-established paradigms. Such scrutiny is essential for science and its application in conservation. However, such reappraisals should be grounded firmly in evidence and scientific principles. We refute a recent article (Wallach and Lundgren 2025) that discounted the role of introduced predators (the domestic cat and red fox) in the extinctions of Australian mammal fauna, finding fault in its premises, analyses, data, and conclusions. Inaccurate and flawed publications have the potential to fuel mis- and disinformation, undermine social license in pest and wildlife management, halt or reverse conservation progress, and ultimately increase the risks of species’ declines and extinctions.

The introduction and spread of cats and foxes have reshaped and significantly diminished Australia’s extraordinary biodiversity and continues to do so. In response to the recognition of the impacts of cats and foxes, a network of continental-scale management, supported by decades of research, is now delivering major conservation outcomes, with enduring ecological and cultural benefits. The dire Australian conservation situation is a window to the broader and ongoing devastating impacts of introduced species on island biota globally (Blackburn et al. 2004). Encouragingly, Australia also serves as a vital demonstration of what conservation can achieve when the factors causing biodiversity decline and extinction are identified and remedied.

## Supplementary Material

biaf204_Supplemental_Files
