# Supplementary material for: Investigating the Causes of an Extinction Catastrophe: Controlling Introduced Predators Remains Essential for Conserving Australia’s Mammals
Source: Bioscience. 2026 Jan 21;76(3):294–307. doi: 10.1093/biosci/biaf204 (PMC13032872; doi:10.1093/biosci/biaf204)
Supplement: biaf204_Supplemental_Files [file biaf204_supplemental_files.zip › Figure_captions_double-spaced.docx]

**Figure captions**

Figure 1. Timing of extirpation of a native mammal population from a location relative to the time of arrival of feral cats at that location. Data are taken directly from Supplement 1 in Wallach and Lundgren (2025), using the last record of the native species, and the earliest arrival date from the range of arrival dates of cats to that location. Based on this information, extirpation of a native mammal occurs *before* cats arrive in only two cases, and these are where Wallach and Lundgren (2025) arbitrarily give the last record dates of 1788 and 1789 (i.e., time of European colonization, in 1788) for undated subfossil material. Of the 178 cases given in the Supplement, eight are omitted from this graph because: they were repeats of another row (n=4), there is no date for the last record of the species (n=1), the species is still present at the location (n=2), or the species never occurred at the location (n=1). Figure prepared by authors.

Figure 2. Timing of extirpation of native mammal populations at a location relative to the arrival of cats and foxes at that location. Estimates (posterior medians and 95% highest density intervals [HDIs]) show prey-level random intercepts for each predator from an interval-censored model accounting for uncertainty in the response variable. Predator averages were 41.1 (95% HDI: 34.9, 47.7) years for cats and 4.1 (95% HDI: -2.5, 11.4) for foxes. Red points (raw data) are the empirical means of the minimum and maximum possible years since the last sighting. Figure prepared by authors.

Figure 3. Graphic of main lines of evidence implicating introduced predators (cats and foxes) in losses of Australian native mammals. Figure prepared by authors.

Figure 4. An example of an endemic mammal species, the burrowing bettong (*Bettongia lesueur*) extirpated from its formerly vast mainland range and now present on the mainland only as populations reintroduced to fenced areas that exclude cats and foxes. Photo credits: top (Robert Lang); bottom left (Andrew Freeman); bottom right (K. Moseby).
