# Supplementary material for: Investigating the Causes of an Extinction Catastrophe: Controlling Introduced Predators Remains Essential for Conserving Australia’s Mammals
Source: Bioscience. 2026 Jan 21;76(3):294–307. doi: 10.1093/biosci/biaf204 (PMC13032872; doi:10.1093/biosci/biaf204)
Supplement: biaf204_Supplemental_Files [file biaf204_supplemental_files.zip › S1_confounded_dating.docx]

**Supplement S1: Examples of errors, omissions or misrepresentations in extirpation database of Wallach & Lundgren**

*Bettongia gaimardi*

Case 4, on mainland (Victoria). A later date of last record (1890) is given in Menkhorst (1995). This later date places extirpation more securely *after* the spread of foxes in Victoria.

*Bettongia lesueur*

Cases 11, 12. The last record given for the state of New South Wales (case 11; 1880) precedes that given in case 12 (1890) for a region of that state.

The well established last record for *Bettongia lesueur* in the south-west Kimberley (1896) (Dahl 1897; McKenzie 1981), post-dating the arrival of foxes, is not given by W&L.

*Bettongia tropica*

Case 20. extirpation from New South Wales is incorrect as the species has never been recorded there (Vernes *et al.* 2023).

*Conilurus albipes*

Case 30. Later dates, of 1861-62 (from nearby), than that used (1846) by W&L are documented by (Menkhorst and Dixon 2023). This later date places extirpation more securely *after* the spread of cats to this region.

*Conilurus capricornensis*

Cases 31 and 32. These are duplicates of the same sole original data point. A date of 1789 is arbitrarily chosen by W&L for subfossil material: that ascription of year is without merit.

*Lagorchestes hirsutus*

Cases 54, 55. A far later last date is given for part of state (case 54) than that given for the whole state (case 55)

*Myrmecobius fasciatus*

Case 130. W&L give a minimum and maximum date for the last record of numbats from Adelaide as 1836, based on a statement in Friend (1990) that ‘numbats were present near Adelaide at the time of its European settlement, but disappeared soon after (Wood Jones 1923)’, and that Adelaide was founded in 1836. This use of 1836 as the last record positions the extirpation as between the earliest and latest given dates of cat arrival, and before the arrival of foxes (1895 to 1905). However, Wood Jones (1923-1925) actually stated that ‘only twenty years ago it was met with along the scrub-lands of the Murray (in South Australia), and earlier than that it existed quite near to Adelaide’. The Murray record is omitted by W&L, but must have been around 1903, certainly post-dating the arrival of cats and almost certainly post-dating the arrival of foxes. The 1836 last date for Adelaide is not supported by the primary source, which noted simply that the last record was ‘earlier than … (1903)’.

*Pseudomys australis*

Case 166. W&L give a last record for this species from Mootwingee region of New South Wales, based on a statement in Lunney (2001) that ‘subfossil remains dated post 1860’ were found in the area. Inexplicably, W&L then give a minimum date of 1840; and they translate post-1860 as 1860, thereby concluding that the extirpation pre-dates the arrival of cats (1870-1880). The timing is moot anyway, as the species is not extirpated from this region, as evident from recent records (Leggett *et al.* 2017).

*Trichosurus vulpecula*

Case 176. Again (as for cases 31 and 32), subfossil material is arbitrarily dated, this time as 1787.

Comprehensive accounts of regional extirpations documented by Kerle *et al.* (1992) are not included. Whereas four of the five cases of extirpation of this species given in W&L putatively pre-date the arrival of foxes, nine of ten regional extirpations described by Kerle and Howe (1992) certainly post-date the arrival of foxes: Flinders Ranges (last records 1926 to 1930s cf. fox arrival 1900-1910), Northern Arid Provine SA (1956, with severe declines reported from sometime after 1935, cf. fox arrival ca. 1910), Finke region Northern Territory (last record late 1940s, cf. fox arrival ca. 1920), Petermann Ranges, Northern Territory (last records 1983, 1989 cf. fox arrival ca. 1925), Central Ranges Northern Territory (last record 1979, cf. fox arrival ca. 1925), Simpson Desert Northern Territory (last record 1959; cf. fox arrival ca. 1920), Sandover and Plenty Rivers region Northern Territory (last record 1980s, cf. fox arrival ca. 1920), Western Desert region Northern Territory (last record uncertain, but declines after 1930s, cf. fox arrival ca. 1920), Tanami Desert Northern Territory (last record 1984; cf fox arrival ca. 1930), South Barkly Northern Territory (only noted from 1905; cf. fox arrival ca. 1925).

**References**

Dahl, K. (1897). Biological notes on on north-Australian mammals. *The Zoologist* **4**, 189-216.

Friend, J. A. (1990). The Numbat *Myrmecobius fasciatus* (Myrmecobiidae): history of decline and potential for recovery. *Proceedings of the Ecological Society of Australia* **16**, 369-377.

Kerle, J. A., Foulkes, J. N., Kimber, R. G., and Papenfus, D. (1992). The decline of the brushtail possum, *Trichosurus vulpecula* (Kerr 1798), in arid Australia. *The Rangeland Journal* **14**, 107-127.

Kerle, J. A. and Howe, C. J. (1992). The breeding biology of a tropical possum, *Trichosurus vulpecula arnhemensis* (Phalangeridae: Marsupialia). *Australian Journal of Zoology* **40**, 653-665.

Leggett, K. E., Welaratne, T., Letnic, M., McLeod, S., and Dawson, T. (2017). Rediscovery of the plains mouse (Pseudomys australis)(Rodentia: Muridae) in New South Wales. *Australian Mammalogy* **40**, 127-130.

Lunney, D. (2001). Causes of the extinction of native mammals of the Western Division of New South Wales: an ecological interpretation of the nineteenth century historical record. *The Rangeland Journal* **23**, 44-70.

McKenzie, N. L. (1981). Mammals of the Phanerozoic South-West Kimberley, Western Australia: biogeography and recent changes. *Journal of Biogeography* **8**, 263-280.

Menkhorst, P. W. (Ed.) (1995). 'Mammals of Victoria: distribution, ecology and conservation.' (Oxford University Press: Melbourne.)

Menkhorst, P. W. and Dixon, J. M. (2023). White-footed rabbit-rat *Conilurus albipes*. In 'Strahan's Mammals of Australia'. (Eds A. M. Baker and I. C. Gynther) pp. 405-406. (Reed New Holland: Wahroonga.)

Vernes, K., Whitehead, T., and Johnson, P. M. (2023). Northern bettong *Bettongia tropica*. In 'Strahan's Mammals of Australia'. (Eds A. M. Baker and I. C. Gynther) pp. 296-298. (Reed New Holland: Wahroonga.)

Wood Jones, F. (1923-1925) 'The mammals of South Australia. Parts I -III.' (Government Printer: Adelaide.)
