# Supplementary material for: Investigating the Causes of an Extinction Catastrophe: Controlling Introduced Predators Remains Essential for Conserving Australia’s Mammals
Source: Bioscience. 2026 Jan 21;76(3):294–307. doi: 10.1093/biosci/biaf204 (PMC13032872; doi:10.1093/biosci/biaf204)
Supplement: biaf204_Supplemental_Files [file biaf204_supplemental_files.zip › Supplement 2_word.docx]

Supplement 2. Extant threatened terrestrial Australian mammal species, as listed under Australian legislation (EPBCA) or in the IUCN Red List. The final column notes whether cats and/or foxes are listed as threats in the IUCN Red Lits accounts or in recovery plans or conservation advices.

| **Scientific name** | **Common name** | **EPBCA** | **IUCN** | **cat or fox listed as threat** |
| --- | --- | --- | --- | --- |
| *Dasyuroides byrnei* | Kowari | EN | VU | YES |
| *Dasyurus geoffroii* | Chuditch, Western Quoll | VU | NT | YES |
| *Dasyurus hallucatus* | Northern Quoll | EN | EN | YES |
| *Dasyurus maculatus* | Spotted-tailed Quoll | all 3 subspp listed | NT | YES |
| *Dasyurus viverrinus* | Eastern Quoll | EN | EN | YES |
| *Parantechinus apicalis* | Dibbler | EN | EN | YES |
| *Sarcophilus harrisii* | Tasmanian Devil | EN | EN | NO |
| *Antechinus argentus* | Silver-headed Antechinus | EN |  | YES |
| *Antechinus arktos* | Black-tailed Antechinus | EN |  | YES |
| *Antechinus bellus* | Fawn Antechinus | VU | VU | YES |
| *Phascogale calura* | Red-tailed Phascogale | VU | NT | YES |
| *Phascogale pirata* | Northern Brush-tailed Phascogale | VU | VU | YES |
| *Sminthopsis butleri* | Butler's Dunnart | VU | VU | YES |
| *Sminthopsis douglasi* | Julia Creek Dunnart | VU | NT | YES |
| *Sminthopsis psammophila* | Sandhill Dunnart | EN | VU | YES |
| *Myrmecobius fasciatus* | Numbat | EN | EN | YES |
| *Isoodon auratus* | Golden Bandicoot | VU (for subspp auratus and barrowensis) | VU | YES |
| *Perameles bougainville* | Western Barred Bandicoot |  | VU | YES |
| *Perameles gunnii* | Eastern Barred Bandicoot | both subspp listed | VU | YES |
| *Macrotis lagotis* | Bilby | VU | VU | YES |
| *Phascolarctos cinereus* | Koala | (VU) | VU | NO |
| *Lasiorhinus krefftii* | Northern Hairy-nosed Wombat | CR | CR | NO |
| *Burramys parvus* | Mountain Pygmy-possum | EN | CR | YES |
| *Gymnobelideus leadbeateri* | Leadbeater's Possum | CR | CR | YES |
| *Petaurus australis* | Yellow-bellied Glider | VU, EN | NT | YES |
| *Petaurus gracilis* | Mahogany Glider | EN | EN | NO |
| *Petauroides volans* | Greater Glider | VU/EN (northern/southern) | VU | NO |
| *Pseudocheirus occidentalis* | Western Ringtail Possum | CR | CR | YES |
| *Bettongia lesueur* | Boodie | all subspp listed | NT | YES |
| *Bettongia penicillata* | Woylie | EN | CR | YES |
| *Bettongia tropica* | Northern Bettong | EN | EN | YES |
| *Potorous gilbertii* | Gilbert's Potoroo | CR | CR | YES |
| *Potorous longipes* | Long-footed Potoroo | EN | VU | YES |
| *Potorous tridactylus* | Long-nosed Potoroo | VU, for both mainland subspp. | NT | YES |
| *Lagorchestes hirsutus* | Rufous Hare-wallaby | all subspp listed | VU | YES |
| *Notamacropus parma* | Parma Wallaby | VU | NT | YES |
| *Onychogalea fraenata* | Bridled Nailtail Wallaby | EN | EN | YES |
| *Petrogale coenensis* | Cape York Rock-wallaby |  | EN | NO |
| *Petrogale concinna* | Nabarlek | sort of all subspp listed | EN | YES |
| *Petrogale lateralis* | Black-footed Rock-wallaby | four subspp. listed | VU | YES |
| *Petrogale penicillata* | Brush-tailed Rock-wallaby | VU | VU | YES |
| *Petrogale persephone* | Proserpine Rock-wallaby | EN | EN | YES |
| *Petrogale sharmani* | Mount Claro Rock-wallaby | VU | VU | NO |
| *Petrogale xanthopus* | Yellow-footed Rock-wallaby | both subspp listed | NT | YES |
| *Setonix brachyurus* | Quokka | VU | VU | YES |
| *Lagostrophus fasciatus* | Banded Hare-wallaby | only extant subsp listed | VU | YES |
| *Crocidura trichura* | Christmas Island Shrew | CR | CR | YES |
| *Pteropus conspicillatus* | Spectacled Flying-fox | EN | LC | NO |
| *Pteropus natalis* | Christmas Island Flying-fox | CR | VU | YES |
| *Pteropus poliocephalus* | Grey-headed Flying-fox | VU | VU | NO |
| *Macroderma gigas* | Ghost Bat | VU | VU | NO |
| *Rhinolophus robertsi* | Greater Large-eared Horseshoe-bat | EN (as R. p. 'large form') | LC | NO |
| *Hipposideros inornatus* | Arnhem Leaf-nosed Bat | EN | VU | NO |
| *Hipposideros semoni* | Semon's Leaf-nosed Bat | VU | LC | NO |
| *Hipposideros stenotis* | Northern Leaf-nosed Bat |  | VU | NO |
| *Saccolaimus saccolaimus* | Bare-rumped Sheath-tailed Bat | listed at subsp level | LC | NO |
| *Phoniscus papuensis* | Golden-tipped Bat |  | VU | NO |
| *Nyctophilus corbeni* | South-eastern Long-eared Bat | VU | VU | NO |
| *Nyctophilus sherrini* | Tasmanian Long-eared Bat |  | VU | NO |
| *Chalinolobus dwyeri* | Large-eared Pied Bat | EN | NT | NO |
| *Falsistrellus mackenziei* | Western False Pipistrelle |  | VU | NO |
| *Conilurus penicillatus* | Brush-tailed Rabbit-rat | VU | VU | YES |
| *Leporillus conditor* | Greater Stick-nest Rat | VU | NT | YES |
| *Mastacomys fuscus* | Broad-toothed Rat | EN (for M. f .mordicus) | NT | YES |
| *Mesembriomys gouldii* | Black-footed Tree-rat | all 3 subspp listed | VU | YES |
| *Notomys aquilo* | Northern Hopping-mouse | EN | EN | YES |
| *Notomys fuscus* | Dusky Hopping-mouse | VU | VU | YES |
| *Pseudomys australis* | Plains Mouse | VU | VU | YES |
| *Pseudomys calabyi* | Kakadu Pebble-mouse |  | VU | YES |
| *Pseudomys fumeus* | Smoky Mouse | EN | VU | YES |
| *Pseudomys gouldii* | Gould's Mouse, Djoongari, Shark Bay Mouse | VU | EX | YES |
| *Pseudomys novaehollandiae* | New Holland Mouse | VU | VU | YES |
| *Pseudomys oralis* | Hastings River Mouse | EN | VU | YES |
| *Pseudomys pilligaensis* | Pilliga Mouse | VU | DD | YES |
| *Pseudomys shortridgei* | Heath Mouse | EN | NT | YES |
| *Zyzomys maini* | Arnhem Rock-rat | VU | VU | NO |
| *Zyzomys palatalis* | Carpentarian Rock-rat | EN | CR | NO |
| *Zyzomys pedunculatus* | Central Rock-rat | CR | CR | YES |
| *Xeromys myoides* | Water Mouse | VU | VU | YES |
